# Purinergic Receptors: Elucidating the Role of these Immune Mediators in HIV-1 Fusion

**DOI:** 10.3390/v12030290

**Published:** 2020-03-07

**Authors:** Tracey L. Freeman, Talia H. Swartz

**Affiliations:** Division of Infectious Diseases, Department of Medicine, Immunology Institute, Icahn School of Medicine at Mount Sinai, New York, NY 10029, USA; tracey.freeman@mssm.edu

**Keywords:** purinergic, HIV-1, fusion, inflammation

## Abstract

Purinergic receptors are inflammatory mediators activated by extracellular nucleotides released by dying or injured cells. Several studies have described an important role for these receptors in HIV-1 entry, particularly regarding their activity on HIV-1 viral membrane fusion. Several reports identify purinergic receptor antagonists that inhibit HIV-1 membrane fusion; these drugs are suspected to act through antagonizing Env-chemokine receptor interactions. They also appear to abrogate activity of downstream mediators that potentiate activation of the NLRP3 inflammasome pathway. Here we review the literature on purinergic receptors, the drugs that inhibit their function, and the evidence implicating these receptors in HIV-1 entry.

## 1. Introduction

### 1.1. HIV and Inflammation

HIV-1 infection is a major cause of morbidity and mortality worldwide, with 37 million individuals living with the disease and 2 million new infections occurring annually [1]. Improved antiretroviral therapy (ART) has significantly increased the life expectancy of people with HIV-1 (PWH). Still, PWH experience chronic inflammation that leads to co-morbidities including cancer, bone disease, and cardiovascular disease [2,3,4,5,6,7,8,9,10,11,12]. The source of this chronic inflammation is multifactorial in nature and includes cell-mediated effects, overproduction of soluble cytokines, coinfections, and immune dysregulation. Increased cell senescence is a primary component of rapid aging and is characterized by CD8^+^ T lymphocyte accumulation and activation [12,13] and secretion of inflammatory mediators that induce chronic low-level inflammation and heightened cell turnover [12,14,15,16]. Desai, et al., characterized multiple progressive sequelae as contributors to HIV-induced accelerated aging: HIV-1 replication in activated immune cells, GI mucosal epithelium depletion with loss of Th17 cells, microbial translocation, thymic dysfunction with loss of naïve T lymphocytes and regulatory CD4^+^ T lymphocytes, and clonal expansion of activated immune cells. This clonal expansion results in the loss of CD28 on T lymphocytes and expedited telomere shortening, ultimately producing nonfunctional, senescent T lymphocytes [10,13,17,18,19,20]. Early infection can also result in fibrosis and dysfunction of lymphoid organs and co-infection with pathogens such as cytomegalovirus (CMV) [21]. Immune exhaustion contributes to inflammation, as HIV-induced PD-1 overexpression on CD4^+^ and CD8^+^ T lymphocytes is not fully normalized by ART [16]. The viral reservoir marks an important mechanism by which inflammation can persist; in lymphoid tissue, HIV-1 productive infection in a small percentage of permissive cells can result in abortive infection of bystander cells (>95% CD4^+^ T lymphocyte population). Bystander cells can undergo pyroptosis, a caspase-1-mediated programmed cell death. Pyroptosis could re-activate more latently-infected cells and attract more uninfected cells to die and sustain chronic inflammation [22,23,24,25,26,27,28]. Finally, soluble biomarkers including d-dimer, hsCRP and IL-6, soluble CD14, and sTNFR II have been associated with comorbidities in large patient cohorts [7,11,29,30,31,32,33,34,35]. These elevated inflammatory biomarkers occur despite ART and do not improve even with treatment intensification [36,37].

### 1.2. Purinergic Receptors

Purinergic receptors have been recognized since the 1970s as mediating an important mechanism of immune function and cell signaling [38,39]. They are widely expressed on mammalian cells. These receptors can detect extracellular nucleotides to activate intracellular signaling events. The receptors are divided into three categories: P1 adenosine receptors, P2X heterotrimeric ATP-gated cation channels, and P2Y G-protein coupled receptors [40,41]. There is a rich literature on the role of the P2X receptor as an important regulator of inflammation and other physiological signaling events [42,43,44,45,46]. The P2X receptors are ATP-gated cation channels. An ATP or other nucleotide agonist binds to the extracellular region of the P2X receptor, inducing a conformational change that opens the receptor central pore, facilitating cation flux [40,47]. Some receptor subtypes have larger pore openings that enable large molecules to pass through [48,49,50]. P2X receptors can undergo rapid desensitization with prolonged ATP exposure, resulting in closure of the channel [51,52]. The subunits of functional P2X receptors can assemble into either homotrimers or heterotrimers; each subunit contains two transmembrane domains, a large extracellular loop with 10 conserved cysteine residues and glycosylation sites, and intracellular N and C termini with consensus phosphorylation sites [41,43,53,54,55]. The P2X7 receptor subtype (P2RX7) is the best characterized in the innate immune response and its role has been well described in inflammatory cytokine signaling, antigen presentation, and lymphocyte proliferation and differentiation [56,57,58]. P2RX7 is activated by sub-millimolar concentrations of extracellular ATP. Physiologically, this ATP is transiently released in the bloodstream in response to acute cell death or injury [40]. P2RX7 has a large pore when assembled as a homotrimer; sustained P2RX7 activation can result in pore opening, enabling passage of molecules up to 900 Da that can induce cell death [43,59]. P2X1 receptors (P2RX1) are expressed at low levels on myeloid cells and lesser so on lymphocytes; fewer reports, therefore, have characterized this receptor with respect to inflammation [60,61,62,63,64,65,66].

P2Y receptors have broad functions across a range of physiological systems including blood clotting, hormonal secretion, vascular responses, cell migration, wound healing, and immune responses [38,39,46,49,67,68,69]. The P2Y receptor subtypes are G protein-coupled receptors that consist of seven transmembrane domains with an extracellular N-terminus and an intracellular C-terminus [67,70,71]. P2Y receptor activation results in G-protein dissociation of α and βγ subunits that induce downstream effector signaling. The P2Y class of receptors consists of a large diversity of sequences that impact different pharmacologic functions [72]. There are eight monomer subtypes. Of these, four (P2Y1, P2Y2, P2Y4, and P2Y6) activate phospholipase C via G_q_, three (P2Y12, P2Y13, and P2Y14) inhibit adenylyl cyclase and activate GIRK-family K^+^ channels through G_i_, and one (P2Y11) triggers intracellular Ca^2+^ and in cAMP levels through both G_q_ and G_s_.

### 1.3. Purinergic Receptors and HIV-1

Purinergic signaling has been demonstrated to be key to the immune response in several infectious processes [73,74,75], including bacterial [76,77,78,79,80], protozoal [81,82,83,84], and viral infections including respiratory viral infections [85,86], hepatitides [63,87,88,89], cytomegalovirus [90], and HIV-1 [91,92,93,94,95,96]. Interestingly, purinergic receptors have been implicated in both immunoprotective and immunopathologic roles in these infection [75,88,97,98]. There is a growing body of evidence that implicates purinergic receptors and both selective and non-selective antagonists of these receptors in HIV-1 infection [99,100,101,102,103,104,105]. Our laboratory and others have explored the role of purinergic receptors in HIV-1 infection and have reported that purinergic receptor antagonists can block HIV-1 infection and reduce inflammatory cytokine production associated with infection [96,106,107]. More specific probing appears to implicate drugs that target purinergic receptors in the inhibition of HIV-1 viral membrane fusion. This will be the subject of the following section.

The first studies that suggested a role for purinergic receptors in HIV-1 pathogenesis took place in the 1980s when suramin was proposed as an anti-retroviral agent and was given to 98 patients with AIDS following in vitro observations of suramin inhibiting reverse transcriptase activity [108]. Suramin was quickly abandoned as a treatment, however, this was due to drug toxicity that resulted in numerous patient fatalities. The fatalities took place in individuals with a high burden of disease and, therefore, suramin’s impact on HIV-1 replication was impossible to assess. Recent investigations focus on the roles of purinergic receptors in HIV-1 pathogenesis and the activities of compounds distinct from suramin.

Since that time, there have been a number of studies that have uncovered roles for purinergic receptors in HIV-1 pathogenesis [91,92,93,94,95,96]. Adenosine receptors have been implicated based on data suggesting an association between CD39 expression and AIDS progression [109]. CD39, an ectoenzyme, generates adenosine that induces purinergic adenosine receptor signaling. The authors observed that inhibition of regulatory T lymphocytes was reduced by CD39 downregulation, resulting in associated elevated levels of T-cell A2A adenosine receptor in HIV-1 infected patients. Additionally, the authors noted that a CD39 polymorphism was associated with reduced CD39 expression and a delay in onset of AIDS.

Sorrell, et al., probed the role of extracellular ATP signaling and observed that treatment with a non-selective P2X antagonist modulated opiate neurotoxicity through HIV-1 Tat [110]. Tovar, et al., observed that HIV-infected macrophages released ATP that reduced dendritic spine density through glutamate receptor downmodulation that was dependent on purinergic receptor signaling [111]. More recently, growing literature has supported the notion of purinergic receptors and their antagonists directly impacting HIV-1 viral replication. Séror, et al., demonstrated that HIV-1 infection induces ATP release in human lymphocytes [95] and that the selective depletion of P2Y2 diminished the HIV-induced inflammatory response. Immunofluorescence indicated that P2Y2 and the ATP channel Pannexin-1 appeared to accumulate at the virological synapse, where an infected donor cell can mediate efficient cell-to-cell transfer to an uninfected cell [112,113].

Hazleton, et al., demonstrated that purinergic receptors play a key role in HIV-1 replication in macrophages through selective pharmacologic inhibition of P2RX1, P2RX7, and P2RY1 [93]. Giroud, et al. described a role for P2RX1 in blocking HIV-1 binding to the chemokine receptors CCR5 and CXCR4 [106]. Swartz, et al., demonstrated that non-selective antagonists of P2X receptors can inhibit HIV-1 infection of CD4^+^ lymphocytes through both cell-to-cell and cell-free mechanisms [96]. Orellana et al., reported that both R5- and X4-tropic HIV-1 infection activates the pannexin-1 ATP channel; this enabled HIV-1 internalization in CD4^+^ T cells [94]. Graziano, et al., demonstrated a P2RX7-dependent phenomenon whereby exposure to extracellular ATP induced rapid release of HIV-1 particles in human monocyte-derived [114]. Numerous studies describe the role of purinergic receptors and their antagonists at various stages of the viral life cycle and downstream impacts on pathogenesis. In the following section, we will review the literature as it pertains to the role of purinergic receptors and their antagonists on HIV-1 fusion.

### 1.4. Purinergic Receptors and HIV-1 Fusion

While a broad range of purinergic receptor antagonists have been demonstrated to inhibit HIV-1 infection [99,100,101,102,103,104,105], only P2RX1-specific compounds have been implicated in HIV-1 membrane fusion. Marin, et al. developed and employed a high-throughput BlaM-Vpr assay that identifies virus-cell membrane fusion via cleavage of a cytoplasmic β-lactamase-fused reporter substrate subsequent to membrane fusion of a β-lactamase-fused virus [115]. A screen of 1280 compounds identified the P2 × 1-antagonizing compounds NF279 and NF449 as inhibitors of virus-cell fusion in the TZM-bl cell line. NF449 and NF279 were further reported as fusion inhibitors in primary monocyte-derived macrophage (MDM), CEM-A, Jurkat, and NP2 cells as measured by the BlaM-Vpr assay [93,106]. Our laboratory developed a novel HIV-1 Cre-lox assay to measure cell-to-cell mediated virus-membrane fusion [96,116,117]. In this assay, transduced Jurkat cells express a reporter cassette containing a lox-P flanked dsRed reporter followed by a Cre-inducible GFP site. Transfected donor Jurkat cells produce HIV-1 that is derived from NL4-3 and packages the Cre Recombinase enzyme (HIV-1 Gag-iCre); HIV-1 Gag-iCre viral fusion with target Jurkat floxRG donor cells releases Cre into the target cell cytoplasm, thereby inducing a red-to-green switch in fluorescence [116,117]. Using this assay, we identified the P2RX1-selective compounds NF279, PPNDS, and NF023 as potent inhibitors of cell-to-cell mediated virus-membrane fusion. The broad-spectrum P2XR antagonist PPADS exhibited partial inhibition of virus-membrane fusion [96].

P2RX7 and P2YR antagonists have been shown to inhibit HIV-1 infection at potencies similar to those of P2RX1 antagonists [93,95,96,107,117,118], but these compounds do not appear to impact virus-cell membrane fusion. The P2RX7 antagonist A740003, which inhibits HIV-1 infection [93], fails to inhibit virus-membrane fusion as measured by BlaM-Vpr assays [93,107,115]. The same assay demonstrated that the broad-spectrum P2 receptor inhibitor PPADS likewise has no effect on virus-membrane fusion, and the Jurkat floxRG assay detected minimal inhibition of fusion inhibition resultant of PPADS treatment [96,115].

Select antagonists of the P2Y class of purinergic receptors have similarly inhibited HIV-1 productive infection in various assays [85,95,105], but there have been no reports of these compounds directly affecting virus-cell fusion. Swartz, et al., using the Jurkat floxRG assay, conducted a screen with a library of 71 purinergic antagonists and found no evidence of fusion inhibition induced by any of the P2YR antagonists [96].

The P2Y11R antagonist NF340, an inhibitor of HIV-1 infection, was assessed as a fusion inhibitor via BlaM-Vpr assays. The compound demonstrated minimal activity as an inhibitor of HXB2, BaL26, and R3A Env-expressing HIV-1 entry in CEM-A or TZM-bl cells, and this minimal effect was seen only at high concentrations [106,115]. The BlaM-Vpr assay was further used to study the P2RY1 antagonist MRS2500 and the P2RY2 antagonist PSB 1114; neither MRS2500 nor PSB 1114 inhibited virus-cell fusion in TZM-bl cells [115].

Giroud et al., employed a temperature-arrested stage (TAS) fusion assay in which prolonged virus-cell incubation at a reduced temperature minimizes the binding efficiency of the Env-coreceptor complex but maintains relative permissivity to CD4-Env binding [106]. The resultant high ratio of Env-CD4 complexes to Env-coreceptor complexes renders subsequent raised-temperature-induced viral fusion more sensitive to inhibition via coreceptor antagonists than to inhibition via CD4 antagonists. When assessed as a TAS fusion inhibitor, NF279 exhibited inhibitory potency that mirrors coreceptor antagonist activity; this potency is distinct from those of CD4 antagonists. A TAS drug-substitution assay revealed that unlike a CD4 antagonist, NF279 fails to inhibit viral fusion to TZM-bl cells at the CD4 binding step. NF279 instead was shown to be interchangeable with coreceptor antagonists in inhibiting fusion. Complicating the hypothesis that NF279 functions as a fusion inhibitor downstream of CD4 binding, employment of this assay with primary MDMs revealed a partial antagonizing activity of NF279 against the Env-CD4 complex in these cells.

A calcium influx assay in TZM-bl cells was used to elucidate the relationship between NF279 and CCR5/CXCR4. Following stimulation with a coreceptor agonist or gp120, NF279 inhibited subsequent cellular calcium influx characteristic of virus-coreceptor binding in a manner similar to that of coreceptor antagonists. This observation, however, was also inconsistent across cell types as NF279 only partially inhibited CXCR4-mediated calcium influx in CEM-A cells. NF279 was also found to inhibit calcium influx induced by a CXCR3/CXCR7 agonist in these cells, highlighting the possibility that NF279 nonspecifically binds to chemokine receptors.

The activity of NF279 was further compared to those of coreceptor antagonists via a BlaM-Vpr assay that assessed BaL26 viral fusion with TZM-bl and JGR.H11 cells expressing a CCR5 mutant (G163R) with reduced binding efficiency for gp120. NF279 as well as the dual CCR5 and CC2b antagonist TAK-779 inhibited virus-cell fusion in both of the CCR5 mutant cell lines; NF279 exhibited ~3-fold increased inhibitory potency against the mutant cell lines relative to wild-type cells. This observation was distinct from CCR5 antagonists Schering C and AD101, both of which failed to inhibit virus-cell fusion in the CCR5 mutant cells. The increased sensitivity of the CCR5 mutants to NF279-mediated fusion inhibition suggests possible competition between NF279 and CCR5 for gp120 binding.

Furthermore, the P2RX1-selective compounds NF279 and NF449 have been shown to inhibit HIV-1 cellular entry with greater activity against X4-tropic virus (HIV-1 HXB2) than with R5- or dual-tropic virus (HIV-1 BaL26 and HIV-1 R3A, respectively) [106,115]. The observation that viral tropism modulates the inhibitory potency of NF449 and NF279 against viral fusion indicates that Env and/or coreceptor mediate purinergic antagonist-dependent fusion inhibition.

Our laboratory additionally demonstrated that NF449 and NF279-mediated fusion inhibition relies on intact gp41 and displays varied activity against diverse clades. The inhibitory activity of NF279 and NF449 appeared to interfere specifically with Env. This was observed as NF279 and NF449 inhibited viral membrane fusion and interfered with PG9 antibody neutralization. PG9 targets the V1V2 region of HIV-1 gp120 [118]. These observations suggested that NF279 and NF449 might act on the V1V2 region, either directly or indirectly.

## 2. Discussion

Despite the efficacy of ART in suppressing HIV-1 replication and maintaining CD4^+^ T lymphocyte counts, PWH experiences numerous comorbidities resultant of HIV-induced chronic inflammation. These inflammatory comorbidities are a prominent concern in HIV-1 treatment as they are an impediment to quality-of-life among PWH and are the leading cause of early mortality among ART-treated PWH.

The immunomodulatory purinergic receptors have been implicated in the immune response against multiple pathogens, and numerous antagonists of these receptors have been shown to abrogate HIV-1 infection in vitro at varying potencies. Select studies have reported reduced expression and/or function of purinergic signaling pathway components as being associated with relatively better outcomes for PWH, but these data are limited and their scope minimal. In assessing the hypothesized clinical applications of P2XR antagonists, our laboratory demonstrated P2XR antagonist-mediated inhibition of both HIV-1 infection and HIV-induced inflammatory cytokine production. These data suggest that P2XR antagonists may represent a novel class of dual-target therapeutics.

Regarding the mechanism of these drugs, the fact that the immunomodulatory purinergic receptors are expressed extracellularly indicates that abrogation of HIV-induced inflammation occurs before HIV-1 cellular entry. Comprehensive screens including multiple classes of purinergic receptor antagonists have identified P2RX1 antagonists, specifically, as inhibitors of cell-virus fusion. P2RX1 antagonists have been demonstrated to inhibit virus-cell fusion in both primary cells and multiple cell lines. This antagonism has been demonstrated through multiple methodologies including the described BlaM-Vpr, Jurkat floxRG, and temperature-arrested state (TAS) fusion assays. Antagonists of the P2RX7 and P2YR receptors, in contrast, were reported as exhibiting minimal or absent activity in inhibiting virus-cell fusion.

Although fusion antagonism appears specific to P2RX1-specific antagonists, these suramin-derived compounds lose receptor subtype specificity at the micromolar concentrations used in these studies [106,107]. Some antagonists have previously been shown to inhibit post-fusion targets, such as the original observation of anti-reverse transcriptase activity by suramin. The documented nonspecific binding of these compounds to multiple diverse targets, paired with the observation that fusion inhibition is only seen with P2RX1 antagonists, renders it unlikely that the inhibition of HIV-1 fusion and infection by these compounds is exclusively resultant of purinergic receptor antagonism.

Because of the low binding specificity of these antagonists at the concentrations required to inhibit HIV-1 infection, it is difficult to assess the precise mechanism by which they inhibit. Numerous studies have, however, identified Env-coreceptor interactions as a likely target of P2RX1 antagonist-mediated fusion inhibition. NF279 has greater inhibitory potency against X4-tropic virus relative to R5-tropic viruses, suggesting that inhibitory activity is dependent on Env and/or coreceptor structure. A TAS assay employed by Giroud, et al. demonstrated that NF279 inhibited fusion at a stage following virus-CD4 attachment and that the kinetics of this inhibition resembled those of coreceptor antagonists [106]. NF279 was additionally shown to inhibit coreceptor agonist-induced calcium influx in a manner similar to coreceptor antagonists.

These observations, however, were inconsistent across cell lines: NF279 was found to only partially inhibit CXCR4-associated calcium influx in CEM-A cells; in this study, NF279 also exhibited inhibitory activity against other chemokine receptor agonists. NF279 was further shown to partially inhibit CD4 attachment in CEM-A cells. These incongruous findings could represent nonspecific binding of NF279 to chemokine receptors, but they may also be resultant of NF279 antagonism of non-coreceptor ion channels such as the various purinergic receptors.

Further probing the mechanism by which the P2RX1 antagonists may inhibit coreceptor engagement, Giroud et al. reported that NF279 exhibits increased inhibitory potency when virus-coreceptor affinity is reduced by a CCR5 mutation, suggesting competition between NF279 and Env for coreceptor binding [106]. Similarly, our group has shown that NF449 inhibits PG9 antibody binding to the V1V2 region of gp120, indicating drug-coreceptor binding competition. Further implicating gp120 in NF449-mediated fusion inhibition, we have reported that NF449 inhibitory activity is dependent on intact gp41. Fusion inhibition is not, however, dependent on gp41 itself, suggesting that gp41-induced structural changes in the conformation of gp120 interfere with NF449-mediated fusion inhibition. Our group has proposed a mechanism in which NF449 either directly binds to the V1V2 region of gp120, blocking coreceptor engagement, or instead induces a conformational change in the V1V2 loop that inhibits coreceptor binding.

Interestingly, NF279 has been widely reported as a more potent inhibitor of HIV-1 infection and membrane fusion than NF449, but NF449 was demonstrated to more potently inhibit PG9-Env binding. This may represent differential binding affinity of the two antagonists to Env and/or additional inhibitory off-target effects of NF279, such as the coreceptor binding suggested by Giroud et al. [106].

Taken as a whole, we suspect that purinergic receptors themselves are not primary mediators of HIV-1 membrane fusion. P2X antagonism via pharmacotherapy, however, likely plays a role in the abrogation of the HIV-induced inflammatory response. It is possible, therefore, that drug-Env binding is the primary mediator of fusion inhibition, while concurrent inhibition of P2X receptors abrogates the HIV-induced cellular inflammatory response. Ultimately, studies investigating the mechanism by which the P2RX1 antagonists inhibit virus-cell fusion and virus-induced inflammatory response are inconclusive due to the non-specificity of these compounds, varied activity among different antagonists, and inconsistent results between cell types.

Based on the collective literature documenting P2RX1 antagonist activity in inhibiting virus-cell fusion, further investigation into the activities and mechanisms of these antagonists is needed. This line of research is particularly relevant to elucidating HIV-1 pathogenesis, as the mechanism by which HIV-1 induces chronic inflammation and its consequential comorbidities remains undefined. Identifying the target of these drugs may provide needed insight into the identification of novel therapeutics that dually mitigate both HIV-1 infection and its resultant chronic inflammation. Developing such dual-target drugs is of critical importance in the treatment of PWH as effective modulation of the HIV-induced immune response is required to enhance the quality-of-life and normalize the lifespan of PWH.
